# Effect of process parameters on vitamins and sensory acceptability in micronutrient‐fortified soymilk prepared by small‐scale batch processing

**DOI:** 10.1002/fsn3.3161

**Published:** 2022-12-05

**Authors:** Dallin M. Hardy, Oscar A. Pike, Bradley J. Taylor, Michael L. Dunn

**Affiliations:** ^1^ Department of Nutrition, Dietetics, and Food Science Brigham Young University Provo Utah USA

**Keywords:** aid, beverage, fortification, humanitarian, soya, storage

## Abstract

Effects of holding time before cooling, cooling method, and light or dark refrigerated storage on the stability of vitamin A, vitamin C, thiamine, riboflavin, and folate were investigated in fortified and unfortified soymilk. Vitamin C loss (6%) and mild vitamin A isomerization occurred when soymilk was held hot after fortification. Cooling bottled soymilk at ambient temperature or in an ice‐water bath did not affect any vitamins. Loss of riboflavin (18%) and vitamin A isomerization occurred during 12 days of light‐exposed refrigerated storage, in contrast to no vitamin degradation during dark refrigerated storage. A sensory panel of youth and children indicated no significant preferences between fortified and unfortified soymilk except for color, where the lighter‐colored unfortified soymilk was preferred. Acceptable vitamin stability and sensory characteristics can be achieved in fortified soymilk produced in small‐scale batch processes with appropriate management of production and storage conditions.

## Graphical abstract

We evaluated the stability of vitamins added to soymilk during small‐scale processing using a “soy cow.” Effects of heating and cooling temperatures and lighted storage were evaluated. Most vitamins were quite stable to the heating and cooling process, but significant degradation occurred in a cabinet mimicking a lighted, refrigerated display.

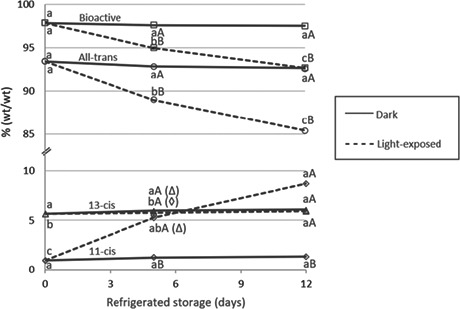

## 1 INTRODUCTION

Soymilk containing high‐quality protein can be produced by minimally trained operators using simple, small‐scale processing equipment (so‐called “soy cows”). Entrepreneurs, charities, and international‐aid organizations have sponsored the placement of such equipment in strategic locations near malnourished populations (Boomgarden, 2016). These systems cook whole soybeans using steam under pressure and then express the product through a manually operated filter press to produce fresh soymilk. The steam‐pasteurized, filtered soymilk is sometimes sweetened and/or flavored and then packaged before refrigerating.

The soymilk thus produced is a good source of protein and calories, but lacks some minerals and vitamins needed to address common nutritional deficiencies. Micronutrient fortification of soymilk prepared in these small‐scale facilities could have a substantial positive impact on the physical/cognitive development and nutritional well‐being of targeted beneficiaries.

While a number of soymilk fortification studies have been published, most of these focused solely on calcium addition and used previously prepared soymilk, purchased at retail or produced in commercial pilot plants (Zhao et al., 2005). Other studies were limited to the bioavailability of different calcium sources added to soymilk, often without further heat processing (Heaney et al., 2000).

Early soymilk fortification studies found that the addition of minerals can cause protein stability problems during thermal processing (Yazici et al., 1997). Rasyid and Hansen (1991) reported that calcium fortification of soymilk was challenging due to the sensitivity of soy proteins to calcium ions. Product coagulation resulted if processing conditions were not carefully controlled. Pathomrungsiyounggul et al. (2007) evaluated different calcium salts in soymilk during lab‐scale processing, finding that product viscosity and sedimentation rate varied depending on the composition.

Thermal processing also leads to vitamin losses in fortified nutritional beverages. Singh et al. (1996) carried out a limited fortification study, adding thiamine, riboflavin, methionine, and lysine, but no minerals, to soymilk, which was then boiled for 5 min or sterilized for 22 min at 15 psi. They found that heat treatment caused substantial loss of both vitamins, compared to unboiled control samples.

In addition to challenges with the physical stability of the product and vitamin stability, the effects of micronutrient addition on soymilk sensory properties must also be considered. Singh et al. (1996) found that higher levels of fortification significantly lowered the flavor acceptability and overall sensory acceptability of soymilk. Yeh et al. (2017) also reported negative sensory effects following the fortification of bovine milk.

Clearly, adding a multivitamin/mineral premix to soymilk produced in small‐scale processing facilities has the potential for achieving significant nutritional benefits. However, simple, reproducible process parameters must be developed to ensure adequate physical and nutrient stability, as well as acceptable sensory properties in the finished product. The objective of this study was to determine sensory properties and the effect of holding time prior to cooling, cooling method, and light or dark refrigerated storage on the stability of vitamin A, vitamin C, thiamine, riboflavin, and folate in fortified and unfortified soymilk produced using small‐scale processing equipment.

## 2 MATERIALS AND METHODS

Emerge‐389 variety soybeans were supplied by Clarkson Grain Company, Inc. Granulated white sugar, noniodized salt, and cooling ice were obtained from a local supermarket. Municipal water was used in water bath processing.

### 2.1 Experimental design

Two batches of soymilk were processed from the same soybean source and all bottling, treatment, and sampling procedures were identical for both production batches. Each batch of soymilk was divided into two lots, one termed “fortified,” which received a premix of micronutrients, sugar, and salt, and the other termed “unfortified,” which received a premix of only sugar and salt. The fortified and unfortified soymilks were then used to conduct four experiments.

The first experiment investigated the effect on vitamin stability of delayed cooling of bulk soymilk. Three subsamples were taken from the bulk soymilk of each lot immediately after premix addition (0 min) and again at 7.5 and 15 min.

The second experiment measured the effect on vitamin stability of cooling bottled soymilk in an ambient‐temperature water bath versus ice‐water bath. At 7.5 min following pasteurization, four 473‐ml clear plastic bottles were filled from each lot (i.e., four fortified bottles and four unfortified bottles), capped with screw cap lids, and two bottles were assigned to each of the two cooling treatments. After cooling as described below, three subsamples were taken from each of the bottles.

Fifteen minutes after premix addition, all remaining soymilk (both fortified and unfortified lots) was bottled in 473‐ml clear plastic bottles, cooled in an ice‐water bath, and refrigerated without light exposure until use in the third and fourth experiments.

The third experiment studied the effect on vitamin stability of light exposure during refrigerated storage of bottled soymilk. Ten bottles of fortified and 10 bottles of unfortified soymilk from each production batch were assigned to each of the two storage regimens: Soymilk bottles were subjected to light exposure for 8 h per day or they were kept in the dark, during refrigerated storage for up to 12 days. Three subsamples from each of the two bottles were taken at baseline (0 days), 5 days, and 12 days of storage. New samples of unopened soymilk bottles were analyzed at all three sampling times to avoid introducing errors through opening, sampling, and reclosing the bottles.

The fourth experiment evaluated the effect on sensory properties of fortified and unfortified bottled soymilk stored refrigerated for no more than 12 days in the dark.

### 2.2 Preparation of premix

A vitamin–mineral fortification mix was obtained from DSM Nutritional Products (Colombia, S.A.). The mix was formulated to meet governmental nutrient content guidelines for the national school feeding program (“Programa de Alimentación Escolar”) of Ecuador, since results from this study were to be implemented in a small‐scale soy‐processing facility in Guayaquil, Ecuador. The mix was intended to deliver 60% of Ecuador's daily recommended intake values (IDR) for vitamin A, vitamin D, vitamin E, vitamin C, thiamine, riboflavin, vitamin B6, niacin, folate, vitamin B12, iron, zinc, copper, and selenium, and 20% of the IDR for calcium per 350 ml portion of fortified beverage. Corn maltodextrin served as the diluent and carrier in the mix. The chemical forms of the fortificants and quantities added per kg mix were as follows: 14.631 g retinol palmitate (250,000 IU/g); 2.439 g cholecalciferol (100,000 IU/g); 42.394 g dl‐alpha tocopherol (500 IU/g); 2.407 g thiamine mononitrate; 1.849 g riboflavin; 2.087 g pyridoxine hydrochloride; 96.015 g sodium ascorbate; 0.252 g folic acid; 19.494 g niacinamide; 1.219 g 0.1% cyanocobalamin; 523.021 g tricalcium phosphate; 0.009 g copper gluconate; 49.792 g 24% ferric pyrophosphate; 0.075 g sodium selenite anhydrous; and 27.317 g zinc sulfate monohydrate.

Fortified and unfortified premixes were prepared 2 days prior to soymilk processing by weighing out the amounts of salt (7.57 g), sugar (804.41 g), and vitamin–mineral fortificant (15.96 g) needed for a single batch of soymilk. All premix components were combined in a 1‐gallon plastic storage bag with ample headspace and shaken in a vigorous circular motion for 3 min to achieve homogeneity. After mixing, bulk premixes were stored at ambient temperature in light‐impermeable packaging.

### 2.3 Soymilk processing

Two batches of soymilk were prepared at the National Soybean Research Laboratory, located at the University of Illinois Champaign‐Urbana, using the facility's standard processing procedures. For each batch of soymilk, 2 kg of dry soybeans were soaked overnight in excess water under refrigeration. Immediately prior to processing, the beans were drained, rinsed, and added to the pressure cooker (Soya Cow Machine, Model No.: SC‐20; SSP Private Ltd.). Water was added to the cooker at a ratio of 8‐part water to 1‐part soybeans (based on the dry soybean weight recorded prior to soaking). The cooker was then closed and steam was injected (with venting until the air was fully displaced with steam – approximately 10 s), whereupon the beans were ground for 2 min. Manually controlled steam pressurization continued until the target temperature (105°C) had been maintained for 2 min (a total of 24 min for batch 1 and 35 min for batch 2). Pressure remained steady at approximately 12 psi (82.7 kPa) for batch 1 and 14 psi (96.5 kPa) for batch 2 for the majority of the processing time. Following processing, the steam valve was shut off and the milk was discharged through a filter into a 23‐L plastic pail.

### 2.4 Premix incorporation

Immediately after collection, each batch of soymilk was divided into two approximately equal lots in tared 23‐L plastic pails and the weight of soymilk in each pail was recorded. The weight of previously prepared premixes was then adjusted as necessary to deliver the following target inputs per kg of soymilk: 0.8 g salt; 80 g white sugar; and 1.687 g DSM fortification mix (fortified soymilk only). Temperatures were recorded with a thermocouple probe immediately prior to premix addition (ranging from 78.6 to 82.0°C), then an appropriate weight of fortified premix was added to one pail (768.30 g for Batch 1 and 773.90 g for Batch 2) while the other pail received an appropriate amount of unfortified premix, to serve as a sweetened, unfortified control (753.02 g for Batch 1 and 758.94 g for Batch 2). The fortified and unfortified premixes were vigorously and simultaneously stirred into each respective treatment lot with a large wire whisk for 1 min. The hot soymilk was then held for one additional min after stirring prior to filling sample bottles to provide at least 2 full minutes of pasteurization time, post‐premix addition. This pasteurization time was intended to accommodate expected cooling and allow for potential variation in final temperatures at different processing facilities. While preliminary evaluation on the benchtop seemed to indicate adequate premix solubility, there was uncertainty around the dispersion and solubility of premix in the larger‐scale batches at NSRL. Therefore, the soymilk was periodically rewhisked during filling the bottles to ensure homogeneity from sample to sample. Subsequent bottle‐to‐bottle analytical results during micronutrient testing did confirm premix homogeneity in the batches.

### 2.5 Packaging, cooling, sampling, and transport

To monitor vitamin degradation in the hot soymilk prior to cooling, three subsamples were taken from bulk soymilk (both fortified and unfortified lots) in 60‐ml glass bottles immediately following the 2 min whisking and pasteurization period, and then again 7.5 and 15 min later. These subsamples were immediately cooled in an ice‐water bath for 30 min before transferring to a freezer.

Immediately after pasteurization, soymilk was filled into plastic 473‐ml polyethylene terephthalate (PET) bottles (6.35 cm diameter), and bottles of both fortified and unfortified soymilk were divided between two cooling treatment baths (ambient vs. ice water), where they were allowed to cool for 30 min. The ambient water bath consisted of 38 L of water at about 26°C prior to sample addition. For both batches, the ambient water bath remained below 29.2°C for the entire cooling process. The ice‐water bath was prepared new for each batch using 19 L of water and 10 kg of ice. Additional ice was added during cooling, and for both batches, the ice water bath measured below 3.5°C prior to sample addition and remained below 7°C for the duration of the cooling process. Following 30 min cooling, sample bottles were immediately refrigerated.

Frozen samples were shipped overnight on dry ice, and refrigerated samples were shipped overnight on ice to Brigham Young University. Upon receipt, and after storage treatments, triplicate subsamples for micronutrient analysis were drawn from each PET bottle and stored in 60‐ml glass bottles at −80°C until analysis. All sample handling during analysis took place under subdued, UV‐filtered lighting to avoid vitamin degradation.

### 2.6 Light‐exposed versus dark storage

Upon receipt, the bottles of soymilk were placed in refrigerated storage at 2°C. Half of the fortified samples and half of the unfortified samples from each batch were exposed to 8 h per day of direct fluorescent lighting, while the remaining samples were stored in the same refrigerator in a light‐protected box.

Light intensity ranged from 1076 to 1281 lux near the top of the bottle, 1012 to 1141 lux near the middle of the bottle, and 969 to 1066 lux at the base of the bottle, depending on the location along the shelf. The position of light‐exposed sample bottles was rotated each day to provide equal light exposure for the duration of refrigerated storage.

On day 0, three subsamples were taken from each of the two fortified and two unfortified bottles from each production batch. On days 5 and 12 of refrigerated storage, three subsamples were drawn from each of the two bottles from each of the four storage treatment groups (fortified or unfortified, light or dark storage) of each batch. All subsamples were stored in 60‐ml glass bottles at −80°C until analysis.

### 2.7 Nutritional analyses

The nutrients selected for analysis were vitamin A, vitamin C, thiamine, riboflavin, and folate. These five vitamins were tested because they are commonly added in enriched and fortified foods and include the most labile water‐soluble (vitamin C) and fat‐soluble (vitamin A) vitamins in the premix (Ball, 2006). Furthermore, vitamin A deficiency and iron deficiency anemia are especially problematic among certain impoverished populations. Therefore, adequate delivery of vitamin A as well as vitamin C and folic acid—the latter two play important roles in iron absorption and red blood cell development, respectively—was important to our fortification objectives (Hallberg et al., 1987; Juarez‐Vazquez et al., 2002). Mineral stability was not tested as minerals were not expected to be labile.

Thiamine and riboflavin were measured using AOAC method 953.17 (AOAC, 2012), with some modifications. Five milliliter of unfortified soymilk or 1 ml of fortified soymilk was pipetted into the flask and weight was recorded. Forty milliliter of 0.1 N HCl was added and the sample was swirled for 1 min before adjusting the pH to 4.5 ± 0.05 with 2.5 M sodium acetate. Next, 500 mg taka‐diastase from *Aspergillus oryzae* (100 U/mg) was added and the flask was swirled, covered with foil, and incubated without agitation at 37°C for 18 h. After incubation, the soy suspension was quantitatively filtered into a 100‐ml volumetric flask and brought to volume. One milliliter was transferred to an amber vial and refrigerated for same‐day riboflavin analysis. Ten milliliter of the remaining filtrate was added to a centrifuge tube with 2.5 g NaCl and swirled until the salt dissolved. Three milliliter of oxidizing reagent (1 ml of 3% potassium ferricyanide brought to 25 ml with 15% NaOH) were then added with gentle swirling. Fifteen milliliter of isobutanol was added and the sample was shaken prior to centrifugation at 1200 *g* for 4 min. One milliliter supernatant was filtered into an amber vial and refrigerated for same‐day thiamine analysis.

Thiamine and riboflavin were quantified against an external standard curve using HPLC equipped with a fluorometric detector (Agilent Technologies, Inc.). Ten microliter sample injections were eluted isocratically using a methanol‐sodium acetate (0.05 M) mobile phase (30:70 v/v) and a flow rate of 1 ml/min. The stationary phase was an octadecyl silane column (Luna C8(2), 150 mm × 4.6 mm, 5 μm particle size, Phenomenex Inc.). Excitation and emission wavelengths were, respectively, 422 nm and 522 nm for riboflavin and 366 nm and 435 nm for thiochrome.

Folate was measured using the AOAC trienzyme extraction method 2004.05 (AOAC, 2012) with slight alterations, including many from Chapman et al. (2010). Rat plasma with anticoagulant factors was used as the folate conjugase enzyme (0.1 ml, male, nonsterile, with lithium and heparin; Pel‐Freeze Biologicals, Catalogue #36161–2, Rogers AR). The folic acid working standard was brought to a concentration of 1 μg/mL, instead of the 10 ng/ml concentration used in the original method. Following filtration, samples were diluted with deionized, autoclaved water by a factor appropriate to approximately equalize the folate concentration between the folic acid standard and all samples.

The 96‐well microtiter plate microbiological assay (*L. casei* ssp. *Rhamnosus*, ATCC #7469) of Tamura (1990) was used with minor modifications. The inoculum was maintained by weekly transfers into fresh lactobacilli broth, followed by 24 hours of incubation at 37°C and then refrigeration until use. Prior to plating the samples, cultures were transferred to depletion media, prepared from lactobacilli broth and folic acid casei media following the method of Chen and Eitenmiller (2007). Absorbance was read using a microtiter plate reader. Data analysis was performed in Excel (Microsoft Corp.) by comparing the absorbance of sample wells from within the linear range of the spectrophotometer with the linear range of the folic acid standard from the same plate (as confirmed by a HorRat value between 0.3 and 1.3).

Vitamin A was measured as all‐trans retinol palmitate and the two degradation isomers with the highest bioactivity, 13‐cis and 11‐cis retinol palmitate. Bioactivity was calculated using the values reported by Weiser and Somorjai (1992). Vitamin A was reported in micrograms of retinol activity equivalents (RAE), calculated using Equation 1.

(1) $$\mathrm{all}-\text{trans}+\left(0.73^{*}13-\mathrm{cis}\right)+\left(0.34^{*}11-\mathrm{cis}\right).$$

The bioactive portion recovered was also reported as a percent of all vitamers as per Equation 2. The all‐trans, 13‐cis, and 11‐cis isomers were also reported as a wt/wt percent of all vitamers without adjusting for bioactivity.

(2) $$\left(\left[\mathrm{all}-\text{trans}+\left(0.73^{*}13-\mathrm{cis}\right)+\left(0.34^{*}11-\mathrm{cis}\right)\right]/\left[\mathrm{all}-\text{trans}+13-\mathrm{cis}+11-\mathrm{cis}\right]\right)*100.$$

For vitamin A analysis, 5 ml soymilk sample was digested as per AOAC Official Method 2012.10 (AOAC, 2012). Samples were then centrifuged for 10 min (1200 *g*) and the supernatant was filtered into an amber vial that was then refrigerated for same‐day analysis. Vitamin A was quantified against an external standard curve using HPLC equipped with a diode array detector (Agilent Technologies, Inc.). Sample injections of 20 μl were eluted as per the gradient elution cycle outlined by McMahon et al. (2013) except that a flow rate of 1 ml/min was necessary to obtain full baseline resolution of the 13‐cis and 11‐cis isomer peaks from each other and the retinol palmitate peak. Mobile phase A was pure hexane and mobile phase B was hexane‐methyl‐t‐butyl ether (75:25 v/v). The stationary phase was amino‐propyl silane bonded to ZORBAX SIL (Zorbax NH_2_ column, 4.6 × 150 mm, 5 μm particle size, Agilent Technologies, Inc.). The detection wavelengths were set at 10 and 325 nm, with reference wavelengths of 100 and 400 nm.

Although there was unexpected variation in vitamin A values from heated soymilk samples, by reporting the quantity of each isomer relative to the sum of all retinol palmitate vitamers, comparisons across treatments were possible.

Vitamin C was analyzed using a method revised by Wang et al. (1988) with modifications based on the commentary of solvent effects by Visser (2012) and the work of Chase et al. (1993). One milliliter of soymilk sample was pipetted into a 25‐ml volumetric flask brought to volume with an acidic digestion solvent (2% trichloroacetic acid, 0.2% dithiothreitol, and 0.05% metaphosphoric acid) and mixed on a stir plate at high speed for 10 min. The sample was centrifuged for 5 min (1600 *g*). The supernatant was filtered into an amber vial and immediately analyzed. Vitamin C was quantified against an external standard using HPLC equipped with a diode array detector (Agilent Technologies, Inc.). Sample injections of 10 μl were eluted isocratically with a sodium acetate mobile phase (0.5 M, pH 4) and a flow rate of 1 ml/min through a Synergi™ Hydro‐pro C‐18 column (250 × 4.6 mm, 5 μm particle size, Phenomenex Inc.). The detection wavelength was 254 nm, with reference wavelengths of 100 and 360 nm.

### 2.8 Sensory evaluation

With the approval of the University's Institutional Review Board (Approval No. 15140), and with parental consent, children aged 7 through 17 were recruited by BYU's Sensory Analysis Laboratory, and the potential panelists were screened for soy allergies and their willingness to try soymilk. Panelists were remunerated $15 for their participation.

A total of 57 children and youth distributed across the recruited age range participated in the sensory panel, consisting of 47% girls and 53% boys. Only 40% of participants reported having had soymilk before. Most panelists (81%) were not sure whether they liked soymilk, and only two panelists stated they disliked soymilk.

Throughout the panel, soymilk was stored on ice in pitchers, and about 75 ml of fortified and unfortified soymilk were poured into 120‐ml clear plastic cups which had been previously labeled with a three‐digit blinding code. Each panelist was presented with two soymilk samples (one fortified and one unfortified), side‐by‐side on the serving tray. Sample presentation from left to right was randomized, and sensory lab workers ensured that the amount of soymilk in each cup was visually similar. A cracker and water were provided to cleanse the palate between samples.

Data were gathered via a digital ballot presented on a computer screen in individually partitioned booths where samples were presented on trays via a pass‐through compartment. Participants answered six questions assessing overall impression, flavor, color, smell, mouthfeel, and aftertaste using a 7‐point hedonic scale with the descriptors: 1 = really bad, 2 = bad, 3 = just a little bad, 4 = maybe good or maybe bad, 5 = just a little good, 6 = good, and 7 = really good. Panelists were also asked to rank the samples in order of preference by selecting which of the two samples they liked best. A final “willingness to consume” question asked how likely they would be to completely drink a full cup of each sample (fortified and unfortified soymilk) in a school cafeteria setting. This question was scored using a 5‐point Likert scale with the following descriptors: 1 = definitely would not drink all of it, 2 = probably would not drink all of it, 3 = maybe drink–maybe not drink all of it, 4 = probably would drink all of it, and 5 = definitely would drink all of it.

### 2.9 Statistical analysis

Data were analyzed using mixed‐models ANOVA (α = .05), blocking by sample bottle and production batch. Paired t‐tests with post hoc Tukey–Kramer adjustment (α = .05) were also performed to determine differences.

Sensory evaluation data from participants who reported a dislike of soymilk were included in the final analysis because the sole exclusion criteria were soy allergy and an unwillingness to try soymilk. Statistical analysis of hedonic scale questions was carried out using one‐way ANOVA with post hoc Tukey's HSD (α = .05). The question asking panelists to rank the samples in order of preference was analyzed using Friedman's analysis of rank. Ballot presentation and statistical data analysis were performed using Compusense 5 software (Compusense Inc.).

## 3 RESULTS

### 3.1 Delayed cooling of bulk soymilk

The temperature of the soymilk gradually dropped at a rate of approximately 1°C per min during the holding period. In unfortified bulk soymilk, there was no significant change in thiamine (0.045 ± 0.003 mg/g), riboflavin (0.018 ± 0.002 mg/g), or folate (15.12 ± 1.21 μg/100 g) held for up to 15 min after pasteurization and before cooling. There was no detectable vitamin C or vitamin A in the unfortified soymilk.

**FIGURE 1:**
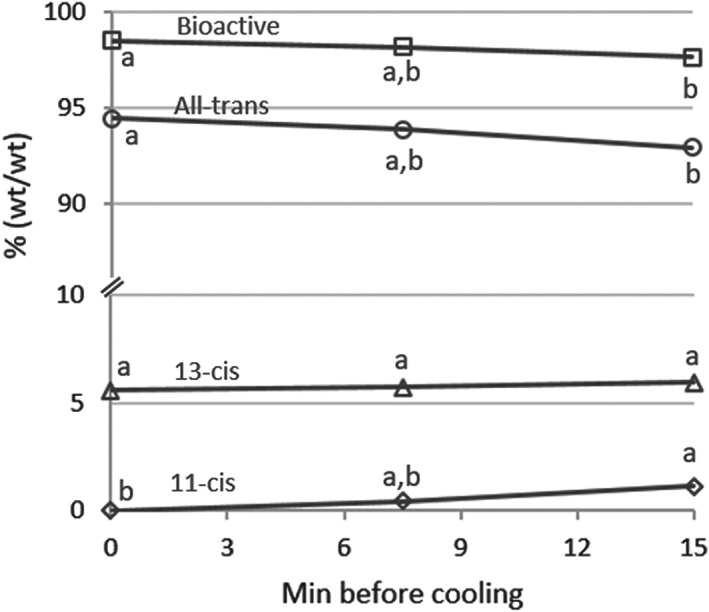
Percentage of vitamin A isomers, as a percent of all vitamers measured, of fortified bulk soymilk held for various times after pasteurization and before cooling. Percentage of bioactive vitamin A is defined as {[All‐trans + (0.73 × 13‐cis) + (0.34 × 11‐cis)] / sum of all vitamers} × 100. Mean ± *SE*. Means for each vitamer with the same letter are not significantly different (*p* > .05)

As shown in Table 1, for fortified bulk soymilk held hot for 15 min after pasteurization and before cooling, there was no significant change in the amounts of thiamine, riboflavin, or folate. There was a 6% decrease in vitamin C for samples held 15 min. The numerical increase in vitamin A for samples held for up to 15 min before cooling was not statistically significant. Figure 1 reports the percentages of vitamin A isomers measured (all‐trans, 13‐cis, and 11‐cis vitamin A) and the resulting percentage of bioactive vitamin A. A 2% degradation of the all‐trans isomer (100% bioactive), primarily to the 11‐cis isomer (which has 34% bioactivity), resulted in a slight (1%) reduction in bioactive vitamin A relative to the sum of all vitamers.

**TABLE 1:** Vitamin content of fortified bulk soymilk held for various times after pasteurization before cooling.

	0 min	7.5 min	15 min
Thiamine (mg/100 g)	0.32 ± 0.02^a^	0.31 ± 0.02^a^	0.33 ± 0.02^a^
Riboflavin (mg/100 g)	0.22 ± 0.01^a^	0.22 ± 0.01^a^	0.22 ± 0.01^a^
Folate (μg/100 g)	60.2 ± 1.27^a^	60.4 ± 1.27^a^	60.5 ± 1.27^a^
Vitamin C (mg/100 g)	12.5 ± 0.14^a^	12.0 ± 0.14^ab^	11.8 ± 0.14^b^
Vitamin A (μg RAE/100 g)	113 ± 10.4^a^	147 ± 10.4^a^	148 ± 10.4^a^

*Note*: ^a,b^Mean ± *SE*. Means in the same row with the same superscript letter are not significantly different (*p* > .05).

### 3.2 Ambient versus ice‐water cooling of bulk soymilk

No differences were found in the quantity of any of the vitamins analyzed in bulk fortified or unfortified soymilk between samples cooled in an ice‐water bath or those cooled in an ambient‐temperature water bath for 30 min after processing. Percentage differences (ambient vs. ice water) were 2.83% for vitamin A, 3.08% for vitamin C, 2.45% for thiamine, 1.33% for riboflavin, and 1.05% for folate—none of which were statistically significant. For further nutritional data on these samples, see Supporting information at https://scholarsarchive.byu.edu/data/43/ (Dunn et al., 2022).

### 3.3 Light‐exposed versus dark storage of refrigerated bottled soymilk

In unfortified bottled soymilk, there was no significant change in thiamine (0.040 ± 0.003 mg/g), riboflavin (0.020 ± 0.001 mg/g), or folate (13.8 ± 0.60 μg/100 g) held up to 12 days in refrigerated storage, whether light‐exposed or in the dark. Again, there was no detectable vitamin C or vitamin A in the unfortified bottled soymilk.

As shown in Table 2 for fortified bottled soymilk, 12 days of refrigerated storage had no statistically significant effect on thiamine, folate, and vitamin C levels, whether light exposed or in the dark during refrigerated storage. However, degradation of riboflavin in fortified, light‐exposed refrigerated storage was significant after 12 days, decreasing from 0.232 mg to 0.190 mg per 100 g of soymilk (18% loss). In contrast, riboflavin loss in dark refrigerated storage was not significant at 12 days, and the difference in riboflavin content between dark and light‐exposed storage was not statistically significant.

**TABLE 2:** Vitamin content of fortified bottled soymilk held in dark or light‐exposed refrigerated storage for up to 12 days.

	Refrigerated storage time		
	0 days	5 days	12 days
Thiamine (mg/100g)			
Dark	0.32 ± 0.01^aA^	0.33 ± 0.01^aA^	0.31 ± 0.01^aA^
Light‐exposed	0.32 ± 0.01^aA^	0.32 ± 0.01^aA^	0.31 ± 0.01^aA^
Riboflavin (mg/100 g)			
Dark	0.23 ± 0.01^aA^	0.23 ± 0.01^aA^	0.22 ± 0.01^aA^
Light‐exposed	0.23 ± 0.01^aA^	0.21 ± 0.01^abA^	0.19 ± 0.01^bA^
Folate (μg/100 g)			
Dark	59.5 ± 0.71^aA^	58.7 ± 0.66^aA^	58.3 ± 0.66^aA^
Light‐exposed	59.5 ± 0.71^aA^	58.5 ± 0.73^aA^	56.7 ± 0.71^aA^
Vitamin C (mg/100 g)			
Dark	10.9 ± 0.56^aA^	10.3 ± 0.55^aA^	10.2 ± 0.55^aA^
Light exposed	10.9 ± 0.56^aA^	9.1 ± 0.56^aA^	8.9 ± 0.50^aA^
Vitamin A (μg RAE/100 g)			
Dark	201 ± 7.6^aA^	187 ± 7.6^aA^	199 ± 7.6^aA^
Light exposed	201 ± 7.6^aA^	185 ± 7.7^aA^	171 ± 7.6^aA^

*Note*: ^a,b,A,B^Mean ± *SE*. Means in the same row with the same lowercase letter superscript are not significantly different (*p* > .05). The two means for dark and light‐exposed treatment in the same column, for each vitamin, with the same uppercase letter superscript are not significantly different (*p* > .05).

**FIGURE 2:**
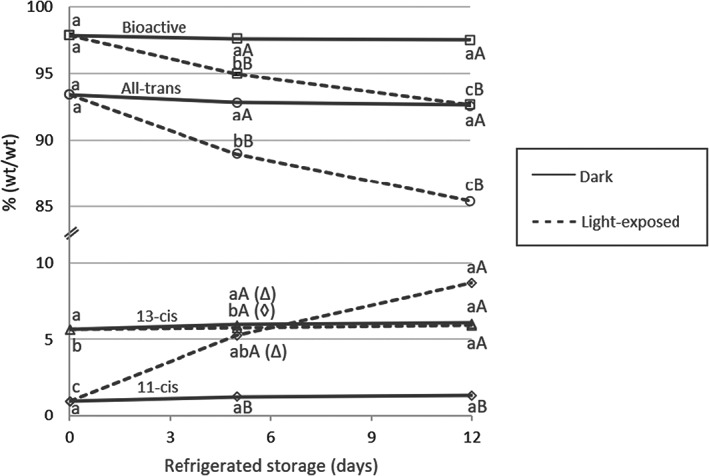
Percentage of vitamin A isomers, as a percent of all vitamers measured, of fortified bulk soymilk held in dark or light‐exposed refrigerated storage for up to 12 days. Percentage bioactive vitamin A defined as {[All‐trans + (0.73 × 13‐cis) + (0.34 × 11‐cis)]/sum of all vitamers} × 100. Mean ± *SE*. Means for each vitamer across storage times with the same lowercase letter superscript (below the solid line for dark and above the dashed line for light exposed) are not significantly different (*p* > .05). The two means for each vitamer for the same storage day with the same uppercase letter superscript (below the solid line for dark and above the dashed line for light exposed) are not significantly different (*p* > .05)

Vitamin A in light‐exposed fortified bottled soymilk was 201 μg RAE/100 g at 0 days and 171 μg RAE/100 g after 12 days of refrigerated storage, but this was not a statistically significant decrease. The difference between light and dark‐stored samples at 12 days was also not statistically significant for vitamin A RAE. However, changes in the percentages of vitamin A isomers were significant, as shown in Figure 2. The percentage of all‐trans isomers significantly decreased after 5 days and again after 12 days in light‐exposed refrigerated storage (5% and 9% loss, respectively, compared to time 0). The difference in all‐trans isomer between light and dark storage at both 5 days and 12 days was also significant. In light‐exposed refrigeration, isomerization favored the 11‐cis product, which increased from time 0 by nearly fivefold at 5 days and by more than eight times by 12 days relative to all measured isomers, while the 13‐cis isomer remained unchanged. The increase of 11‐cis isomer in light‐exposed samples was significant compared to dark‐stored samples at 5 days and 12 days. By contrast, the formation of 11‐cis isomer in dark refrigerated storage was not significant even after 12 days, while 13‐cis isomer content increased significantly after 12 days of dark storage (8% increase relative to all vitamers). 13‐cis isomer formation in light‐exposed storage was not significant, and the difference between light and dark treatments was not significant for the 13‐cis isomer at either storage period. The net result of all isomerization, as measured by the bioactivity‐adjusted sum relative to the non‐adjusted sum of all vitamers reported, was a significant loss of vitamin A activity from baseline in light‐exposed storage at both 5 days (3%) and 12 days (5%), which was also significantly different from dark storage.

### 3.4 Sensory evaluation of bottled soymilk

Due to the difficulty of recruiting an adequate number of children and youth, which requires accompaniment by a parent or guardian, our panelist numbers were limited. However, we were able to recruit 57 panelists, which is more than the minimum number recommended by Stone and Sidel (2004) and Meilgaard et al. (2007).

As shown in Table 3, there was no significant difference in mean sensory scores between unfortified and fortified soymilk except for color, where the panelists scored the unfortified soymilk slightly higher. Mean panelist scores were 5.0 (“just a little good”) or above for all attributes except “aftertaste,” which was scored at 4.5 for unfortified and 4.6 for fortified soymilk. The mean rating for overall likability, the first question asked, was 5.2 for both samples. Regarding overall likeability, 79% of responses were positive (i.e., “just a little good,” “good,” or “really good”) for both the fortified and unfortified soymilk. When asked the final question “If you were served a full cup of this sample in your school cafeteria,  would you DRINK all of it or not?,” 42% of the panelists responded they “probably” or “definitely” would drink all the fortified soymilk and 37% responded they “probably” or “definitely” would drink all of the unfortified soymilk. Again, the responses were not significantly different between fortified and unfortified samples (see Supporting information at https://scholarsarchive.byu.edu/data/43/).

**TABLE 3:** Sensory panelist ratings of unfortified and fortified bottled soymilk.

Attribute^†^	Unfortified soymilk	Fortified soymilk
Overall likability	5.2 ± 1.4^a^	5.2 ± 1.6^a^
Flavor	5.0 ± 1.6^a^	5.1 ± 1.6^a^
Color	6.0 ± 1.0^a^	5.6 ± 1.2^b^
Smell	5.1 ± 1.2^a^	5.0 ± 1.4^a^
Mouthfeel	5.6 ± 1.4^a^	5.8 ± 1.5^a^
Aftertaste	4.5 ± 1.8^a^	4.6 ± 1.8^a^
Willingness to consume^‡^	3.0 ± 1.2^a^	3.0 ± 1.3^a^

*Note*: ^a,b^Mean ± *SE*. Means in the same row with the same superscript letter are not significantly different (*p* > .05).

^†^ Numbers represent a 7‐point hedonic, where 1 = really bad, 2 = bad, 3 = just a little bad, 4 = maybe good or maybe bad, 5 = just a little good, 6 = good, and 7 = really good.

^‡^ Numbers represent a 5‐point Likert scale, where 1 = definitely would not drink all of it, 2 = probably would not drink all of it, 3 = maybe drink–maybe not drink all of it, 4 = probably would drink all of it, and 5 = definitely would drink all of it.

Fifty‐six percent of panelists ranked the fortified soymilk as being preferred over the unfortified soymilk, but this was not significantly different from the 44% of panelists who ranked unfortified soymilk as preferred.

## 4 DISCUSSION

That there was no difference in thiamine, riboflavin, and folate levels between soymilk cooled immediately after fortification and soymilk left hot for 15 min prior to cooling is evidence that delivery of these three nutrients is robust even in less‐than‐ideal processing conditions. We believe that the addition of the premix at the lower temperature of 80°C, compared to the previously discussed ~99°C pasteurization used by Singh et al. (1996), was probably the main factor leading to the stability of these vitamins during processing in our study.

A significant portion of the fortified vitamin C was lost due to heat exposure. Most of the loss seemed to occur at the highest temperatures immediately after fortification (two‐thirds of the total average decrease between premix addition and 15 min post‐fortification occurred within the first 7.5 min), suggesting that the degradation would be difficult to avoid even if considerable efforts were made to rapidly bottle and cool the product. Sharma and Lal (2005) found that added microencapsulated vitamin C in pasteurized (30 min at 63°C) and sterilized (15 min at 121°C) buffalo milk decreased by 12% and 44%, respectively, indicating that time–temperature relationships are significant with vitamin C degradation. Since the sodium ascorbate used in the fortification premix was not protected by microencapsulation, the observed loss of vitamin C is not surprising. Despite the loss, however, fortification of soymilk in the present study still increased the vitamin C content of soymilk, from an undetectable level in unfortified soymilk to more than 10 mg/100 ml in the fortified product prior to storage. Given that the dosages involved are still well below established tolerable upper levels of acceptable intake, and that loss of vitamin C occurs and cannot be entirely prevented with rapid cooling, increased vitamin C overage in the premix (e.g., from 50% to 130% of current) to account for the loss in the hot soymilk after fortification would be acceptable and warranted.

Impressively, the vitamin A used in our experiment (water dispersible; 250,000 IU/g) was only slightly unstable prior to cooling. It is possible that the water‐solubilizing matrix used to deliver the retinol palmitate protected vitamin A from degradation. Also, it may be that this matrix also made it difficult to extract the vitamin A for analysis, until it had been exposed to temperatures above 60°C for 15 min, cooled, and placed into storage. Conversion to less bioactive isomers did occur after the fortified soymilk had been left hot for 15 min. However, the degradation did not appear to be practically significant, and surprisingly, increases in 13‐cis isomer reported by others during heat treatment of foodstuffs (Kurzer, 2013) were not observed. The proportion of 11‐cis isomer relative to all isomers significantly increased 15 min after premix addition, but less than 2% of the all‐trans isomer and only 1% of the bioactive portion were lost. This suggests that, at least for the type of vitamin A ingredient used in the fortification premix, the majority of vitamin A activity is preserved in this small batch soymilk production process, even without expedited bottling and cooling after fortification.

Most of the vitamins did not show a significant decline during refrigerated storage of fortified soymilk, although some downward trends were apparent. Thiamine, folate, vitamin C, and vitamin A RAE had no statistically significant degradation after 12 days in dark or light‐exposed refrigerated storage, although the latter three showed a downward trend during light‐exposed storage. Although statistical significance was not reached within the 12 days of storage in our study, degradation of the vitamins exhibiting a downward trend may have been significant under stronger lighting, longer light exposure, or longer storage—conditions that could exist in a lighted, refrigerated display case of a supermarket. This suggests the importance of minimizing the time to consumption of the fortified beverage. Okwunodulu and Iwe (2015) reported a slow but steady loss of vitamin C during ambient‐temperature storage of fortified, sprouted soymilk. Regarding stability, folate may have been resistant to degradation during both prolonged heat exposure and light‐exposed storage due to the protective effect of polysaccharides (including any fiber present) against light, heat, and oxygen, as reported by Liu et al. (2012) and Ding and Yao (2013).

In contrast to the other vitamins, riboflavin did not decline during dark storage but did decrease by 18% during light‐exposed storage. The sensitivity of riboflavin to light and high temperatures is well documented (Sheraz et al., 2014). The current study confirms similar reports of riboflavin's sensitivity to light‐exposed soymilk (Bianchi et al., 2015).

The numerical 15% decrease in vitamin A RAE during 12 days of light‐exposed refrigeration is of concern. Statistical significance may have been observed had there been less variation in the data, which may be attributable to the experimental variables, as explained above. In terms of each isomer as a percent of total retinol palmitate vitamers measured, vitamin A degradation was apparent. Not only was there a significant decrease in the proportion of fully bioactive all‐trans retinol palmitate during light exposure, the 11‐cis isomer, with inferior bioactivity (34%), was produced preferentially over the more bioactive (73%) 13‐cis isomer. This result confirms reports by Jung et al. (1998) and Chen et al. (1996) that the formation of isomers other than 13‐cis (all of which have lower bioactivity) predominates under conditions of light‐exposed storage while 13‐cis isomer formation is favored in the dark. As noted by Panfili et al. (2008), there is some error inherent in the interpretation of vitamin A degradation when not all isomers are measured; however, this observation does not change the practical significance of all‐trans retinol palmitate being degraded in light‐exposed storage.

In summary, our results indicate that significant loss of vitamin A and riboflavin could occur within 5–10 days with even, moderate, non‐continuous light exposure, and suggests loss of vitamin C and folate could also become significant in a setting of high‐intensity, long‐duration light exposure such as might occur in a well‐lit store display. Given the degradative trends observed in light‐exposed refrigerated storage for at least some of the vitamins measured, strategies for reducing nutrient loss are of interest if micronutrient‐fortified soymilk must be exposed to light in storage or during consumer display. Work by Jung et al. (1998) and Kim et al. (2000) suggests that fortification with vitamin C and/or an array of other vitamins is protective against vitamin A isomerization. However, this strategy is already inherent in our fortification setting due to the comprehensive nature of the micronutrient premix. Furthermore, it may not be effective in the presence of fortificant minerals, in particular iron and zinc (Pinkaew et al., 2012). Given that these two minerals are among the nutrients most critical for at‐risk populations, removing them from the premix in an attempt to reduce vitamin loss is not desirable. For the vitamins (except folate) that trended toward degradation in light‐exposed storage, a large portion of the degradation seemed to occur within the first 5 days, suggesting that managing inventory so as to decrease the time between production and consumption may be effective. If that is not viable, it is recommended that the product be held in the dark for as long as possible before putting it in a display cabinet. Protective packaging could also be a promising option. For instance, Bianchi et al. (2015) demonstrated that sufficient light‐protective additives in HDPE plastic bottles were able to protect soymilk against sensory changes for up to 15 days and riboflavin degradation for up to 29 days compared to clear plastic controls. Obviously, avoiding light exposure between processing and the point of consumption would be ideal. Despite significant 13‐cis isomer production in dark refrigerated storage, no significant loss of bioactive vitamin A occurred. Neither was there a significant loss of other vitamins when stored in the dark.

The magnitude of the standard deviations for the sensory results indicates that there was considerable variability in responses from our young panelists, indicating that a larger number of panelists may be useful for this age category. Nevertheless, our results indicate that micronutrient‐fortified soymilk was generally acceptable to children and youth, with none of the means below the midpoint of 4 on the 7‐point hedonic scale. No preferences for unfortified soymilk over fortified soymilk were exhibited by the panelists for overall likeability, or any of the organoleptic properties evaluated except color. More importantly, willingness to consume the soymilk was not negatively affected by fortification; panelists were as likely as not to consider consuming a full serving of both the unfortified and fortified soymilk. This result is encouragingly consistent with the results of the sensory study by Reilly et al. (2006), in which, after 43 weeks of menu availability alongside dairy milk, 22.3% of students in three US elementary schools voluntarily chose soymilk and consumed an average of 58% of each carton. Given that in neither study children were likely to be driven to choose soymilk due to excessive hunger and, in the case of Reilly et al. (2006), excellent alternatives were available, even higher consumption may reasonably be expected in a humanitarian or community nutrition setting.

The fortified soymilk had a distinctly yellow hue (likely due to the riboflavin and folate in the fortification premix), which made the unfortified soymilk look lighter and whiter in the side‐by‐side panel comparison. However, while the preference for the color of unfortified soymilk over fortified soymilk was statistically significant, the practical significance of this preference is questionable. In fact, the color of soymilk, fortified or unfortified, seemed to be one of its most appealing properties because color received the highest mean score of all the organoleptic properties evaluated and the standard deviation was relatively small. (No individual panelists ranked the color of either soymilk sample as “bad” or “very bad,” while both fortified and unfortified samples were rated as “bad” or “very bad” by one or more panelists for every other organoleptic property evaluated.) Given that 65% of the panelists rated the fortified soymilk color either “good” or “really good,” it is clear that the color of the fortified soymilk was acceptable and that the color of the fortified sample was viewed negatively only in contrast to the unfortified soymilk—a comparison less likely to be made in a real‐world setting of soymilk consumption.

The only other organoleptic characteristic approaching statistical differentiation between the two samples was mouthfeel (*p* = .185), with the average score for fortified soymilk being slightly more positive than for the unfortified samples. The fortified samples were slightly thicker, possibly due to the corn starch diluent, which made up nearly 25% of the fortification premix by weight. Another likely explanation for the slight increase in thickness was the potential interaction of added minerals, such as calcium, with the soy proteins. Saeidy et al. (2014) reported increased viscosity in soymilk due to calcium fortification and used microencapsulation and chelation of calcium to help reduce viscosity. Given that the slight increase in viscosity was not significant and did not negatively affect sensory scores, it does not appear necessary to alter the fortificant to preserve the texture of the unfortified soymilk. There was no evidence of extensive protein–calcium coagulation, as observed by Pathomrungsiyounggul et al. (2007) and others, as described in the Introduction. Although we confirmed that significant thickening can occur with our fortification premix at higher inclusion rates, the quantity of calcium we added (7.13 mM) was about one‐third of the level added by Pathomrungsiyounggul et al. (2007), and the form of calcium in our premix (tricalcium phosphate) was that recommended by these authors for minimization of calcium coagulation.

Willingness to consume soymilk is likely the biggest obstacle in supplementing a child's diet with micronutrients through this means. It is difficult to predict how sensory results generated under controlled conditions with children in the United States might compare to those of children in less‐developed countries, but populations in need of supplemental nutrition may be more willing to consume soymilk if it were offered to them. Local customs, flavor/sweetness, and cultural preferences would need to be considered to enhance the acceptability and liking while balancing caloric contribution to the total diet. Additionally, esthetic packaging and appetite‐conducive distribution settings may enhance humanitarian and community nutrition programs.

## 5 CONCLUSION

Micronutrient enrichment of soymilk is feasible in small‐scale batch production settings, such as those operated by small businesses and humanitarian agencies in developing nations implementing localized interventions. Key observations were as follows: (1) ambient‐temperature water bath cooling (rather than ice‐enabled temperature reduction step) allows sufficient vitamin retention, and (2) most vitamins are retained despite delayed cooling mimicking the slow, nonmechanized, and often minimally staffed settings of small‐scale batch processes. Therefore, even when the populations to be served lacked the facilities and manpower required for optimal production, including rapid bottling and cooling, “soy cows” can be expected to provide a desirable nutritional beverage. An increased overage of vitamin C could counter thermal losses of this cost‐effective vitamin during processing. The other vitamins evaluated did not undergo rapid degradation during 12 days of dark refrigerated storage. A light‐protected product of similar composition and processing should deliver the desired nutritional profile and acceptable shelf life. The demonstrated possibility of delivering substantial quantities of essential micronutrients in just 375 ml of soymilk with practically no compromise in organoleptic acceptability indicates that fortified soymilk as a standalone intervention could help address micronutrient intake in targeted populations.

## CONFLICT OF INTEREST

The authors report no conflict of interest.

## ETHICAL APPROVAL

This study was approved by the Institutional Review Board of Brigham Young University. ID 15140.

## INFORMED CONSENT

Written informed consent was obtained from all study participants.

## Supporting information


TableS1‐S4

## Data Availability

The data that support the findings of this study are openly available in Scholars Archive at https://scholarsarchive.byu.edu/data/43/, reference number [ScholarsArchive ISSN: 2572‐4479].
