# Supplementary material for: Effect of process parameters on vitamins and sensory acceptability in micronutrient‐fortified soymilk prepared by small‐scale batch processing
Source: Food Sci Nutr. 2022 Dec 5;11(3):1272–82. doi: 10.1002/fsn3.3161 (PMC10002871; doi:10.1002/fsn3.3161)
Supplement: Supplementary file 1 — TableS1‐S4 [file FSN3-11-1272-s001.docx]

Supplemental Table 1. Vitamin content of unfortified bulk soymilk held for various times after pasteurization before cooling.

|  | Time after pasteurization and before cooling | | | | |
| --- | --- | --- | --- | --- | --- |
|  | 0 min |  | 7.5 min |  | 15 min |
| Thiamine (mg/100g) | 0.045±0.003^a^ |  | 0.043±0.003^a^ |  | 0.046±0.003^a^ |
| Riboflavin (mg/100g) | 0.018±0.002^a^ |  | 0.017±0.002^a^ |  | 0.019±0.002^a^ |
| Folate (μg/100g) | 15.12±1.21^a^ |  | 15.63±1.21^a^ |  | 15.76±1.24^a^ |
| Vitamin A and Vitamin C were not detected in unfortified samples.  ^a^ Mean ± SE. Means in the same row with the same superscript letter are not significantly different (P > 0.05). | | | | | |

Supplemental Table 2. Vitamin content of micronutrient fortified soymilk cooled in ambient temperature water or in an ice water bath.

|  | Ambient |  | Ice |
| --- | --- | --- | --- |
| Thiamine (mg/100g) | 0.327 ±0.011^a^ |  | 0.335 ±0.011^a^ |
| Riboflavin (mg/100g) | 0.226 ±0.009^a^ |  | 0.223 ±0.009^a^ |
| Folate (μg/100g) | 60.85 ±1.17^a^ |  | 61.49 ±1.14^a^ |
| Vitamin C (mg/100g) | 12.03 ±0.45^a^ |  | 11.66 ±0.45^a^ |
| Vitamin A (μg RAE/100g) | 152.76 ±7.68^a^ |  | 157.08 ±7.68^a^ |
| % All-trans (%, wt/wt) | 93.53 ±0.20^a^ |  | 93.42 ±0.20^a^ |
| % 13-cis (%, wt/wt) | 5.57 ±0.07^a^ |  | 5.600.07^a^ |
| % 11-cis (%, wt/wt) | 0.90 ±1.19^a^ |  | 0.99 ±0.19^a^ |
| % Bioactive (%, wt/wt) * | 97.90 ±0.13^a^ |  | 97.84 ±0.13^a^ |
| ^a^ Mean ± SE. Means in the same row with the same superscript letter are not significantly different (P > 0.05). *Percentage of recovered retinyl palmitate with biological vitamin A activity, defined as: {[All-trans + (0.73*13-cis) + (0.34*11-cis)] / sum of all vitamers} x 100 | | | |

Supplemental Table 3. Vitamin content of unfortified soymilk cooled in ambient temperature water or in an ice water bath.

|  | Ambient |  | Ice |
| --- | --- | --- | --- |
| Thiamine (mg/100g) | 0.043 ±0.003^a^ |  | 0.046 ±0.003^a^ |
| Riboflavin (mg/100g) | 0.018 ±0.002^a^ |  | 0.019 ±0.002^a^ |
| Folate (μg/100g) | 16.32 ±0.62^a^ |  | 16.65 ±0.57^a^ |

Vitamin A and Vitamin C were not detected in unfortified samples.

^a^ Mean ± SE. Means in the same row with the same superscript letter are not significantly different (P > 0.05).

Supplemental Table 4. Differences in panelist responses to the question: “If you were served a full cup of this sample in your school cafeteria, would you DRINK all of it or not?” for micronutrient fortified vs. unfortified soymilk.

| Sample | 1=definitely would not drink all of it | 2=probably would not drink all of it | 3=maybe drink – maybe not drink all of it | 4=probably would drink all of it | 5=definitely would drink all of it | Total |
| --- | --- | --- | --- | --- | --- | --- |
| Unfortified | 5 | 16 | 16 | 14 | 6 | 57 |
| Fortified | 6 | 18 | 9 | 16 | 8 | 57 |

Supplemental Table 5. Panelist rankings of micronutrient fortified and unfortified soymilk samples in order of preference.

|  | Ranked as 1^st^ Preference | | |  | Ranked as 2^nd^ Preference | | |
| --- | --- | --- | --- | --- | --- | --- | --- |
| Sample | # of panelists |  | (%) |  | # of panelists |  | (%) |
| Fortified | 32 ^a^ |  | (56.1) |  | 25 ^a^ |  | (43.9) |
| Unfortified | 25 ^a^ |  | (43.9) |  | 32 ^a^ |  | (56.1) |
| ^a^ Numbers in the same column with the same superscript letter are not significantly different (P > 0.05). | | | | | | | |
